# An FPGA Platform for Next-Generation Grating Encoders

**DOI:** 10.3390/s20082266

**Published:** 2020-04-16

**Authors:** Yaodong Han, Kai Ni, Xinghui Li, Guanhao Wu, Kangning Yu, Qian Zhou, Xiaohao Wang

**Affiliations:** 1 Shenzhen International Graduate School, Tsinghua University, Shenzhen 518055, China; hyd17@mails.tsinghua.edu.cn (Y.H.); ni.kai@sz.tsinghua.edu.cn (K.N.); guanhaowu@tsinghua.edu.cn (G.W.); yukn18@mails.tsinghua.edu.cn (K.Y.); zhou.qian@sz.tsinghua.edu.cn (Q.Z.); wang.xiaohao@sz.tsinghua.edu.cn (X.W.); 2 Department of Precision Instrument, Tsinghua University, Haidian District, Beijing 100084, China

**Keywords:** grating encoder, diffraction, interferometry, FPGA, real-time, multi-axes

## Abstract

Among various nanometer-level displacement measurement methods, grating interferometry-based linear encoders are widely used due to their high robustness, relatively low cost, and compactness. One trend of grating encoders is multi-axis measurement capability for simultaneous precision positioning and small order error motion measurement. However, due to both lack of suitable hardware data processing platform and of a real-time displacement calculation system, meeting the requirements of real-time data processing while maintaining the nanometer order resolutions on all these axes is a challenge. To solve above-mentioned problem, in this paper we introduce a design and experimental validation of a field programmable gate array (FPGA)-cored real-time data processing platform for grating encoders. This platform includes the following functions. First, a front-end photodetector and I/V conversion analog circuit are used to realize basic analog signal filtering, while an eight-channel parallel, 16-bit precision, 200 kSPS maximum acquisition rate Analog-to-digital (ADC) is used to obtain digital signals that are easy to process. Then, an FPGA-based digital signal processing platform is implemented, which can calculate the displacement values corresponding to the phase subdivision signals in parallel and in real time at high speed. Finally, the displacement result is transferred by USB2.0 to the PC in real time through an Universal Asynchronous Receiver/Transmitter (UART) serial port to form a complete real-time displacement calculation system. The experimental results show that the system achieves real-time data processing and displacement result display while meeting the high accuracy of traditional offline data solution methods, which demonstrates the industrial potential and practicality of our absolute two-dimensional grating scale displacement measurement system.

## 1. Introduction

Grating encoders, a specific type of linear encoder, are key components for precision positioning in various industrial engineering applications. Due to their high accuracy, robustness against environmental variances, relatively low cost, and compactness, grating encoders now hold more than 75% market share among various precision positioning solutions. Depending on the measurement method, there are two trends of grating encoders; one is single axis ultra-high precision and the other is multi-axis [1,2,3,4,5,6]. For high precision applications, the diffraction beam interferometry principle is utilized. This method is also known as interference scanning, and employs a fine grating with a micrometer-order period. Nanometer-scale resolution can be achieved for commercial products and sub-nanometer resolution in laboratory systems. For multi-axis solutions, grating encoders can provide both positioning accuracy and small motion error, usually including translational and rotational motion, which is beyond the capabilities of commercial planar encoders that allow simultaneous measurement of X- and Y-direction displacements.

So far, many researchers have provided effective methods, spanning from two-axes to six-axes [7,8,9,10,11,12,13,14,15,16,17]. For multi-axes measurement, a combination of translational with rotational motion measurement is used. For multi-axes translational measurement, a two-axis scale grating with a micrometer-order grating period is employed and the X- and Y-direction diffraction beams interference with each other, allowing simultaneous measurement of two-axes translational motion [6,7,9,11,12,13,14]. The incident direction of the two orthogonal beams can be along the grating normal or in a Littrow configuration [10,15,16]. The in-plane two-axes measurement is effectively expanded to out-of-plane Z-direction measurement by adding grating of the same scale, also known as a reference grating, and configured in a traditional Michelson optical layout [11,14]. On the other hand, for multi-axes rotational measurement, an improved laser autocollimator is employed by introducing a diffraction beam from the scale grating [7,8,10,16]. This beam will change its propagation direction when there is a roll motion. Since these light beams for translational and rotational measurement can be arranged along a common path, the encoder’s structure can be relatively compact and measurement stability can be ensured [7,10,16,17,18].

The feasibility of multi-axes measurement has been demonstrated in several studies. However, it should be noted that most of these solutions were validated in an off-line manner, and all data were processed using a PC-based system [19]. There are few studies analyzing real-time data processing methods for newly-developed grating encoders [20,21,22]. Indeed, a four-step optical layout-based multi-axis displacement calculation algorithm and a multiple quadrant photo detector (QPD)-based multi-axis angle algorithm usually require simultaneous processing of around more than ten signals at high speed, which is challenging in terms of both hardware and programing. For solving this problem and to bridge the gap between lab prototype solutions and a product that can benefit most industrial applications, we investigate an alternative approach for real-time data processing. First of all, as mentioned above, the widely-used method in the above-mentioned multi-axis grating encoders is off-line data processing using MATLAB, where several channels of interference signals detected by photoelectric detectors are eventually converted to voltage signals that are easy to be analyzed. However, this method limits the real-time processing capabilities of the measurement platform and hinders the practicality of real-time display of displacement measurement results [23,24].

One trend of optical encoders is miniaturization by integrating encoder components on a single chip, which can also achieve cost reduction [25]. In order to realize a high-precision, high-speed, low-latency, parallel, large-data-quantity displacement real-time computing platform, we first consider solutions that include Advanced RISC Machines (ARMs) like single chips, high-performance central processing units (CPUs), and Field Programmable Gate Arrays (FPGAs) [23,26]. Compared to ARMs and high-performance CPUs, FPGAs have the advantage of parallel computing, which can realize the parallel acquisition of eight signals and multi-module parallel computation. Besides, FPGA is often used in the laboratory level signal processing platform, which has real-time performance and can measure multi-dimensional signals at the same time, such as 4D (3D + t) interference microscopy [27]. In consideration of FPGA’s low cost and the order of mHz, there are also some smart sensors combining optical principles and FPGA technology to achieve high precision measurement [28]. In addition, the front-end circuit noise and environmental noise also need to be filtered, and the core algorithm of the high-order filter is convolution, so FPGA’s parallel raw data filtering capabilities are more convenient. In terms of latency, although FPGA clock frequency is not as high as that of commercial CPUs, their clock is at the level of several hundred MHz (ns-level clock), which has already met the low latency requirements of this platform [29,30,31]. In addition, the FPGAs’ internal circuit structure and pins can be reprogrammed, with flexible and rich resource extensions, and it is easy to implement subsequent structural and algorithm optimization. Thus, FPGAs are as suitable as the hardware platform to realize high-precision, high-speed, low-latency, parallelization, and large data displacement measurements [32,33]. Therefore, in this paper we present a method using FPGA to implement existing optical algorithms into the circuit structure. Using Analog-to-Digital Converters (ADCs), we process the data in parallel through the FPGA hardware platform, calculate the displacement values and transmit them to the PC in real time through the serial port to realize the real-time display of displacement measurement.

The following Section explains the principle of the grating reading head. The third section presents the design of the algorithm and FPGA circuit structure. The fourth section describes the experimental platform and the results. In Section 5, we discuss the results and compare them with traditional scale displacement calculation methods.

## 2. Principle

**Figure 1:**
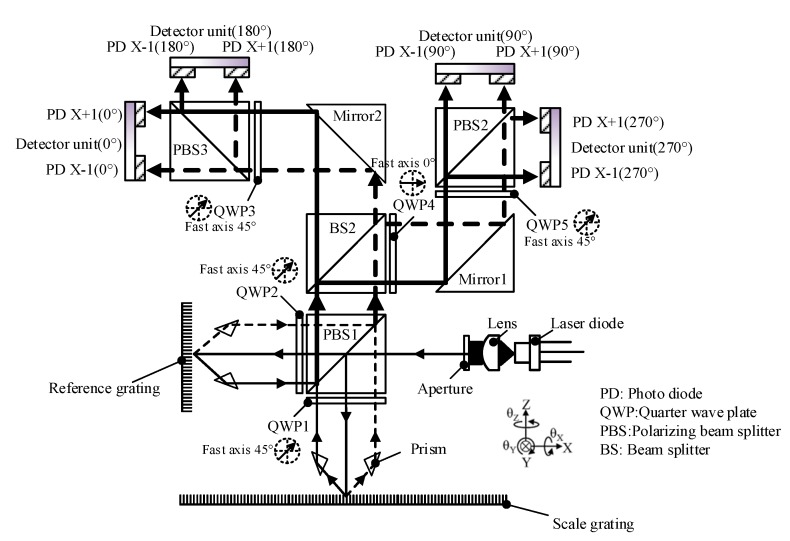
A typical optical layout of a grating encoder for which the proposed platform is designed.

Figure 1 shows our laboratory’s optical layout of a multi-axis grating encoder, for which the real-time data processing platform was designed. It consists of a reading head and a scale grating that are mounted on a stage base and moving element, respectively, for detecting the displacement of the moving element. The scale grating is usually a two-axis one, i.e., it includes the same grating types in orthogonal directions, usually in a one-micron scale. It should be noted that, for simplicity, in this manuscript we use a one-axis grating for explanations. The one-axis grating can provide in-plane X-direction positioning and an out-of-plane Z-direction error motion detection, and this can be easily expanded to in-plane X- and Y-directions using two-axis scale gratings.

The reading head is composed of a coherent laser diode (LD) as the light source, while a grating having the same parameters with the scale grating is used as a reference and is called the reference grating. The beam from the LD is split using a polarizing beam splitter (PBS) into P- and S-polarization beams, which then propagate towards the scale and reference gratings, respectively. Between the PBS and the gratings, quarter-wave plates (QWPs) are placed with fast axis at 45° to the polarization direction. In this manner, the two sets of two first-order diffraction beams from the two gratings change their polarization states and completely pass through and are reflected by PBS1, which greatly improves the power usage. After this, the four first-order diffraction beams are projected to a four-step optical layout, where BS2 divides the diffraction beams in two halves and QWP 4 generates a 90° phase delay for the half projected onto PBS2. Mirrors 1 and 2 here are used to change propagation direction so that these optics can be reconfigured if necessary. QWPs 3 and 5 are used to change the p-and s-polarization into left- and right-circular polarization so than they can be further divided by PBSs 2 and 3. By using the four sets of interference signals with a 90° phase delay, the DC components of the eight interference signals can be eliminated and the motion direction can be determined.

As mentioned above, the light beam generated by LD is divided into p-line and s-line polarized light through BS1, and is incident on a reference grating and a diffraction grating through a quarter wave plate. The measurement beam *Us_α_* generated at the measurement grating and the diffraction beam *Ur_α_* generated at the diffraction grating pass through the two quarter-wave plates again and converge at PBS1. After passing through BS2, a part of the diffracted beam passes through QWP4, QWP5, and PBS2 in this order and is received by the photodetector (PD) to obtain 90° and 270° ± 1 level interference signals.

Therefore, the intensity of the interference signal detected by PD (90°) and PD (270°) are:

(1) $$I_{\alpha }{}_{\left(90{}^{\circ}\right)}=U_{\alpha }{}_{\left(90{}^{\circ}\right)}\cdot \bar{U_{\alpha }{}_{\left(90{}^{\circ}\right)}}=\frac{1}{2}U_{0}^{2}\cdot \left\{1+\cos\left(\Omega_{\alpha }+\Phi \right)\right\},$$

(2) $$I_{\alpha }{}_{\left(270{}^{\circ}\right)}=U_{\alpha }{}_{\left(270{}^{\circ}\right)}\cdot \bar{U_{\alpha }{}_{\left(270{}^{\circ}\right)}}=\frac{1}{2}U_{0}^{2}\cdot \left\{1-\cos\left(\Omega_{\alpha }+\Phi \right)\right\},$$

where *Uα* represents the combined light intensity. The other part of the diffracted beam passes through BS2, QWP3, and PBS3 in that order and is received by the PDs, which is considered as ±1 level interference signals at 0° and 180°.

So, the intensities of the interference signals detected by PD (0°) and PD (180°) are:

(3) $$I_{\alpha }{}_{\left(0{}^{\circ}\right)}=U_{\alpha }{}_{\left(0{}^{\circ}\right)}\cdot \bar{U_{\alpha }{}_{\left(0{}^{\circ}\right)}}=\frac{1}{2}U_{0}^{2}\cdot \left\{1+\sin\left(\Omega_{\alpha }+\Phi \right)\right\},$$

(4) $$I_{\alpha }{}_{\left(180{}^{\circ}\right)}=U_{\alpha }{}_{\left(180{}^{\circ}\right)}\cdot \bar{U_{\alpha }{}_{\left(180{}^{\circ}\right)}}=\frac{1}{2}U_{0}^{2}\cdot \left\{1-\sin\left(\Omega_{\alpha }+\Phi \right)\right\},$$

From the above equation, we see that the amplitude and DC components of the four interference signals *I_α_*_(0°)_, *I_α_*_(90°)_, *I_α_*_(180°)_, and *I_α_*_(270°)_, are all related to *U*_0_^2^, and the phase differences of the four interference signals are 0°, 90°, 180°, and 270°. For the ±1 level diffracted beam, the DC component can be eliminated using the following operations:

(5) $$S_{X+1}=\frac{I_{X+1}\left(0{}^{\circ}\right)-I_{X+1}\left(180{}^{\circ}\right)}{I_{X+1}\left(0{}^{\circ}\right)+I_{X+1}\left(180{}^{\circ}\right)}=\sin\left(\Omega_{\alpha }+\Phi \right),$$

(6) $$S^{\prime }{}_{X+1}=\frac{I_{X+1}\left(90{}^{\circ}\right)-I_{X+1}\left(270{}^{\circ}\right)}{I_{X+1}\left(90{}^{\circ}\right)+I_{X+1}\left(270{}^{\circ}\right)}=\cos\left(\Omega_{\alpha }+\Phi \right),$$

(7) $$S_{X-1}=\frac{I_{X-1}\left(0{}^{\circ}\right)-I_{X-1}\left(180{}^{\circ}\right)}{I_{X-1}\left(0{}^{\circ}\right)+I_{X-1}\left(180{}^{\circ}\right)}=\sin\left(-\Omega_{\alpha }+\Phi \right),$$

(8) $$S^{\prime }{}_{X-1}=\frac{I_{X-1}\left(90{}^{\circ}\right)-I_{X-1}\left(270{}^{\circ}\right)}{I_{X-1}\left(90{}^{\circ}\right)+I_{X-1}\left(270{}^{\circ}\right)}=\cos\left(-\Omega_{\alpha }+\Phi \right),$$

Combining Equations (5)–(8), we obtain the displacement of the scale along the X- and Z-directions, which are:

(9) $$\Omega_{\alpha }+\Phi =\tan^{-1}\left(\frac{S_{X+1}}{S^{\prime }{}_{X+1}}\right),$$

(10) $$-\Omega_{\alpha }+\Phi =\tan^{-1}\left(\frac{S_{X-1}}{S^{\prime }{}_{X-1}}\right),$$

According to the above equation, it is possible to calculate the displacement of the grating in the X-direction, and the magnitude of the jitter in the Z-direction:

(11) $$\Delta x=\frac{g\Omega }{2\pi }=\frac{g}{4\pi }\left\{\tan^{-1}\left(\frac{S_{X+1}}{S^{\prime }{}_{X+1}}\right)-\tan^{-1}\left(\frac{S_{X-1}}{S^{\prime }{}_{X-1}}\right)\right\},$$

(12) $$\Delta z=\frac{\lambda \varphi }{2\pi \left(1+\cos\theta \right)}=\frac{\lambda }{4\pi \left(1+\cos\theta \right)}\cdot \left\{\tan^{-1}\left(\frac{S_{X+1}}{S^{\prime }{}_{X+1}}\right)+\tan^{-1}\left(\frac{S_{X-1}}{S^{\prime }{}_{X-1}}\right)\right\}.$$

where *λ* is the laser wavelength, *g* is the groove width of the grating, and the scale is 1 μm.

## 3. Data Processing Algorithm Design

**Figure 2:**
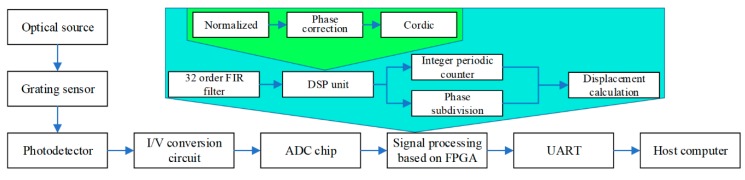
The overall process design of the displacement calculating platform.

The schematic design for the hardware platform is shown in Figure 2. First, the laser beam passes through the grating reading head sensor described above to generate eight interference signals, which are received by the PDs. Then, the analog circuit at the front end converts and converts the mA-level current signal to a V-level voltage signal. In the next step, the eight-channel analog signals are converted into digital signals by the ADC chip, which are then input to the FPGA in parallel for digital signal processing.

The FPGA is our core platform for algorithm implementation. The main algorithm first filters the noise-containing signals through a 32-order Finite Impulse Response (FIR) filter in order to facilitate subsequent calculations. Then, the filtered output enters the digital signal processing module, which mainly includes three steps: first, we normalize the eight-channel filtered signals to convert the unequal-amplitude signals into equal-amplitude signals. Due to errors in the manufacturing process of the reading head, the obtained signal will add a certain phase shift to the original phase. Therefore, a phase correction module is used to correct the phase shift signal to the ideal 0°, 90°, 180°, and 270° signals. Finally, arctangent phase demodulation is performed and a COordinate Rotation DIgital Computer (CORDIC) algorithm is used to calculate the true phase information. After that, the whole phase counting and scattered period calculation are performed on the existing phase information. The whole period calculation method is to count the number of peaks and the scattered phase calculation method is Equation (11), described above. The next module is Binary-Coded Decimal (BCD) transcoding, which converts hexadecimal to decimal numbers that are transmitted to the PC through a Universal Asynchronous Receiver/Transmitter (UART). This allows real-time calculation and display of the displacement.

**Figure 3:**
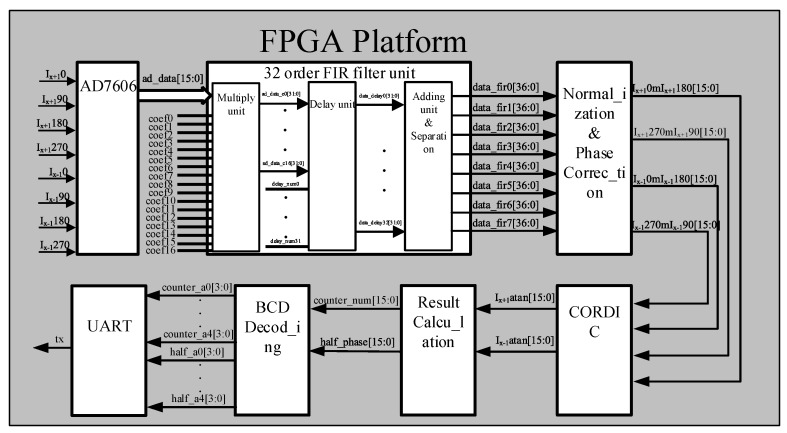
Field programmable gate array (FPGA) platform design.

The specific implementation block diagram is shown in Figure 3. In this paper, an AD7606 analog-to-digital conversion chip is used, and a drive algorithm is applied to implement parallel sampling of eight-phase analog signals at a sampling rate of 100 kHz with a sampling accuracy of 16 bits. We use a 32-order FIR filter to remove the noise of the sampled signal. The principle is to convolve the original signal and the filter impulse response constants. The equation is as follows:

(13) $$y\left(n\right)=x\left(n\right)*h\left(n\right)=\sum_{k=0}^{N}h\left(k\right)x\left(n-k\right).$$

where *x*(*n*) is the input signal, *h*(*n*) is the impulse response, that is, the tap coefficient, and *N* is the order of the FIR filter, which means that the tap number is *n* + 1. In order to fully apply the advantages of FPGA in implementing the FIR algorithm, we choose a three-stage pipeline, which reduces the delay and ensures the real-time performance of data calculation and demodulation. The three-stage pipeline algorithm is as follows:

Stage 1: the original signal and tap coefficients are multiplied, where only half of the multiplications are necessary since the FIR filter coefficients are symmetric;

Stage 2: Apply FIFO (First In First Out) to buffer the data after multiplication to ensure the eight-way signal alignment;

Stage 3: Sum the delayed data for the subsequent separation of eight useful signals. This completes the application of the 32-order FIR filter algorithm.

The original signal is affected by the interference intensity of the optical signal and the amplification effect of the front-end analog circuit, resulting in different amplitudes. For the accuracy of subsequent calculations, we applied a normalization algorithm, implemented by first storing the eight filtered signals in parallel into the FIFO, then setting a certain depth to obtain enough useful signal data. Then, we read the data in the FIFO and determine the maximum absolute value of the filtered signal in real time. After that, all output signals are divided by the maximum absolute value.

After analyzing the normalized signals, we found that the phases of the four signals of the same interference level do not have a strict difference of 90° difference. The reason is that the grating reading head causes a phase error due to glue sticking or stress release during the installation process. This kind of error is difficult to eliminate in the process, so we implemented an algorithm on the FPGA circuit platform to correct the phase error in real time. The normalized real signal equation is as follows:

(14) $$S_{X+1}=\sin\left(\Omega_{\alpha }+\Phi \right),$$

(15) $$S^{\prime }{}_{X+1}=\cos\left(\Omega_{\alpha }+\Phi -\sigma \right),$$

Compared with the theoretical signals, as shown in Equations (5) and (6), real signals do not have exactly a 90° difference, but have a phase error, as shown in Equation (15). So, we implemented a hardware algorithm to eliminate the phase error and correct the normalized signal in real time. The required signal calculation equation is as follows:

(16) $${\left(S_{X+1}\right)}_{\mathrm{co}rrected}=\cos\left(\Omega_{\alpha }+\Phi \right)=\frac{\cos\left(\Omega_{\alpha }+\Phi -\sigma \right)-\sin\left(\Omega_{\alpha }+\Phi \right)\sin\left(\sigma \right)}{\cos\left(\sigma \right)}.$$

Taking *S_X+1_* as the signal of reference, to obtain the corrected signal (*S_X_*_+1_)*_corrected_*, the same three-stage pipeline is applied: one-stage multiplication, one-stage subtraction, and one-stage division with the IP core to implement the phase correction algorithm.

After obtaining the four phase-corrected signals, the arctangent values of the ±1 levels need to be calculated separately. This algorithm uses CORDIC’s arctangent IP core and inputs two 16-bit sine and cosine signals. The output arctangent phase value is also 16-bit from −π to +π. Then, we use the method of threshold determination to count the peak number of the whole period of the arctangent value, determine the number of changes in the whole period caused by the scale displacement, and then apply Equation (11) to calculate the phase of the scattered period in real time. The output result is a 16-bit fixed-point number. Then the result is BCD decoded, that is, the hexadecimal is converted into a decimal number that can be displayed by PC. The classic “greater than 4 shift plus 3” algorithm is used to ensure the low delay of the decoding module in the form of a combination circuit. Finally, we designed the Universal Asynchronous Receiver/Transmitter (UART) serial port driver timing code, the baud rate and the UART frequency-divided clock, and set the parity bit to prevent slipping and errors, and transmit the result to the PC for display correctly and in real time.

The above is the main module of the FPGA hardware platform algorithm design of this paper. The Register Transfer Level (RTL) netlist structure was synthesized, and MODELSIM was used for timing simulation. Pin and timing constraints and layout were performed, and CHIPSCOPE was used to debug the chip. A signal generator was used as the waveform input to verify the correctness of the algorithm. Finally, we combined the FPGA platform, the optical platform and other units to validate the system performance.

## 4. Results

**Figure 4:**
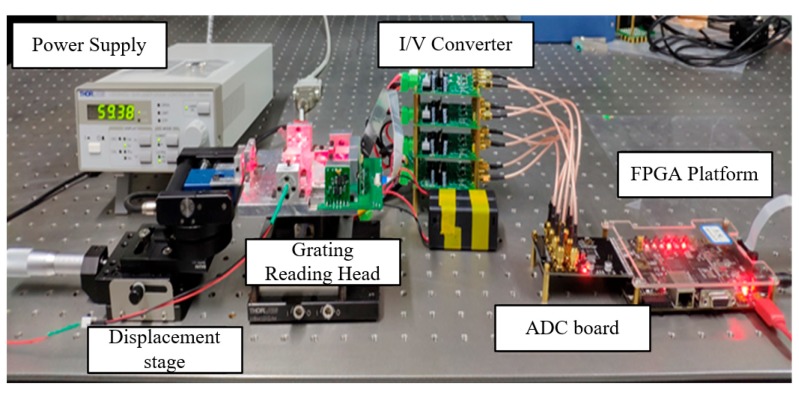
Hardware platform and experimental device.

The experimental equipment and devices are shown in Figure 4. From left to right are the laser light source, the precision stage, the grating reading head, the 8-channel I/V conversion circuit, the ADC chip, and the FPGA board. The power was set to about 60 mA to excite the photodiode and generate a 660 nm laser beam to irradiate the grating reading head. When the precision stage was displaced in the X direction, eight phase difference signals were generated. The eight optical signals were received by the photodetector and the small current signal was transmitted to the I/V conversion circuit, converted into a voltage signal and amplified, followed by simple low-pass filtering. Then, the eight analog voltage signals were input to the AD7606 chip, whose 40-pin interface was connected to the FPGA development board. Finally, the calculated displacement value is transmitted to the PC through the UART interface on the board.

**Figure 5:**
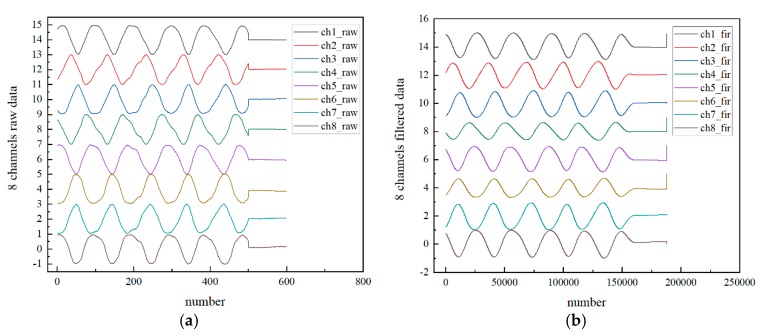
(**a**) Data acquired by AD and (**b**) FIR-filtered data.

We programmed the designed algorithm file, turned on the displacement stage, power supply, analog circuit, FPGA platform, and other hardware, connected the PC and opened the serial port for experimental analysis. The results of each part are shown in Figure 5. Figure 5a shows the data chart after recording of the eight channels using the AD7606. It is clear that the original data contains some signal noise, which causes the signal itself to not be smooth, inhibiting subsequent digital signal processing. After analyzing the noise signal, we found that our useful signal frequency is from 10 Hz to 100 Hz and the noise is mainly low frequency. So we chose low pass filter so that we could get the specific parameters for FPGA by MATLAB’s filter designer tool. Figure 5b shows the signal filtered by the 32-order FIR filter. Compared with Figure 5a, it can be clearly seen that the original noise is eliminated, the signal is smooth, and the effect is significant.

In order to eliminate the DC component in the phase-subdivided signal, as shown in Equations (5)–(8), the original ±1 level interference signal needs to be subtracted separately to obtain four channels to be calculated using the arctangent algorithm. However, as mentioned above, since the real signal is different from the ideal signal, it is not strictly a 90° phase difference value, so phase correction is required.

**Figure 6:**
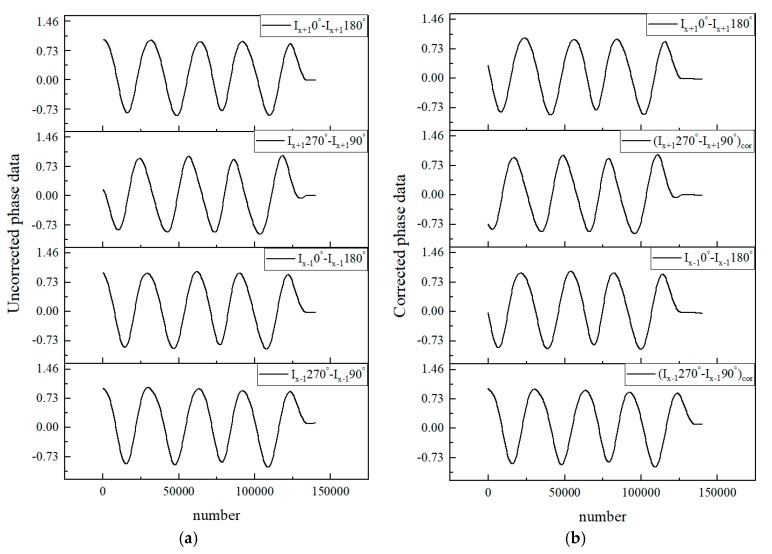
Uncorrected phase data (**a**) and corrected phase data (**b**).

Figure 6a is the signal before phase correction. For the +1 level signals *I_x_*_+1_(0°) − *I_x_*_+1_(180°) and *I_x_*_+1_(90°) − *I_x_*_+1_(270°), it can be seen that the phase difference basically meets the requirements, i.e., it is close to 90°; but for −1 level signals *I_x_*_−1_(0°) − *I_x_*_−1_(180°) and *I_x_*_−1_(90°) − *I_x_*_−1_(270°), it can be seen that the phase difference of the original signal is close to 0°. Theoretically, the former must lead the latter by a 90° phase difference, otherwise the accuracy of subsequent arctangent calculation will be greatly affected. Therefore, as shown in Figure 6b, phase correction is performed using Equation (16). After correction, the new *(I_x_*_+1_(90°) − *I_x_*_+1_(270°))*_corrected_* and *(I_x_*_−1_(90°) − *I_x_*_−1_(270°))*_corrected_* values are obtained. It can be seen that every two signals of the ±1 levels have a strict phase difference of 90°. The result is consistent with the ideal waveform, and the effect is significant.

**Figure 7:**
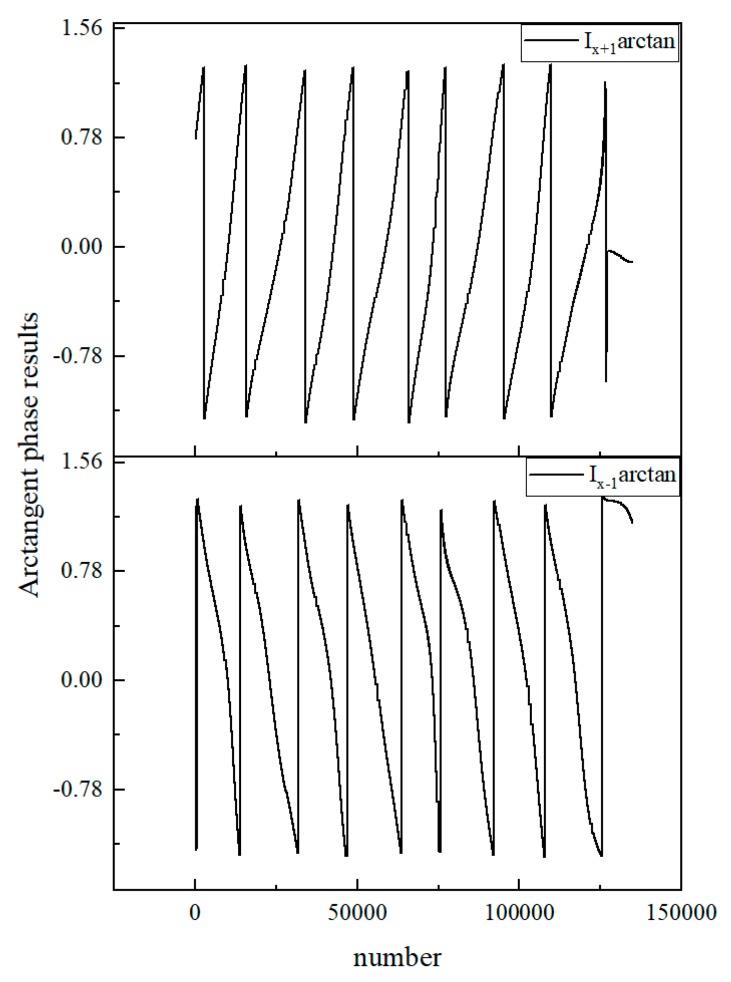
Positive level’s and negative level’s arctangent phase results.

After obtaining the four corrected signals, we need to find the arctangent value of the two channels separately. We used the Xilinx CORDIC IP core to realize the arctangent algorithm. The original result range was −π ~ π, but in order to meet the subsequent algorithm requirements, we converted it to −π/2 ~ π/2. The final arctangent results are shown in Figure 7 below.

After calculating the arctangent phase value of the ±1 levels interference signals, the displacement in the X-direction can be determined using Equation (11), and the displacement results are analyzed, as shown in the following Table 1:

## 5. Discussion

In this paper, the high-speed, parallel, and erasable circuit structure of an FPGA is utilized to realize the design and implementation of a high-precision, high-speed real-time data processing platform. In view of the lack of real-time calculations in traditional scale displacement measurement platforms, the traditional offline data solution method was improved, and a substantial contribution to the practicality and industrialization of scale displacement measurement is made.

By analyzing the results and comparing the displacement accuracy between the FPGA online real-time platform and offline displacement calculation method using MATLAB, we see that the absolute error of the results obtained using the FPGA hardware platform is less than 0.006 μm, which meets the requirements of this paper. In addition, as for real-time performance, when the FPGA clock frequency is 50 MHz, the maximum delay time of the FPGA algorithm is 13 ms, which also meets the real-time requirement. If the delay must be further reduced, more advanced FPGA chips can be used to achieve a clock frequency of thousands of MHz, and the algorithm delay time can be less than 1 ms.

For our future work, we will explore achieving lower latency and higher bit accuracy using more powerful FPGA hardware platform. In addition, the core algorithm will be encapsulated, pins can be derived and optical custom IP cores will be generated, which can increase the portability and versatility of the algorithm. Furthermore, a universal circuit structure for the optical displacement platform will be designed.

## Figures and Tables

**Table 1:** Comparative analysis of offline result obtained using MATLAB and FPGA results obtained online.

Matlab Offline Result/μm	FPGA Online Result/μm	Absolute Error/μm
1.1972	1.2023	+0.0051
2.1587	2.1624	+0.0038
3.1932	3.1917	−0.0016
4.1948	4.1968	+0.0020
5.2298	5.2291	−0.0007
6.1969	6.1959	−0.0010
7.1683	7.1724	+0.0041
8.2164	8.2191	+0.0027
9.2116	9.2107	−0.0009
10.2018	10.2033	+0.0015
